# Tetra­aqua­(2,2′-bipyridine-κ^2^ *N*,*N*′)magnesium(II) bis­(4-fluoro­benzoate)

**DOI:** 10.1107/S1600536810047690

**Published:** 2010-11-24

**Authors:** Bi-Song Zhang, Jian-Ping Qiu, Li-Hua Liu, Wei Xu

**Affiliations:** a College of Material Science and Chemical Engineering, Jinhua College of Profession and Technology, Jinhua, Zhejiang 321017, People’s Republic of China; b Municipal Key Laboratory of Inorganic Materials Chemistry, Institute for Solid State Chemistry, Ninbo University, Ningbo 315211, People’s Republic of China

## Abstract

The title compound, [Mg(C_10_H_8_N_2_)(H_2_O)_4_](C_7_H_4_FO_2_)_2_, consists of a bivalent [Mg(C_10_H_8_N_2_)(H_2_O)_4_]^2+^ cation and two 4-fluorbenzoate anions. In the complex cation, the Mg^II^ atom is coordinated by two N atoms from a 2,2′-bipyridine ligand and four water O atoms in a distorted MgN_2_O_4_ octa­hedral geometry. The Mg^II^ atom is located on a twofold rotation axis and thus a cation exhibits *C*_2_ mol­ecular symmetry. The 2,2′-bipyridine ligands exhibit nearly perfect planarity (r.m.s. deviations = 0.0061 Å). In the crystal, O—H⋯O and C—H⋯O hydrogen bonds link the cations and anions into a three-dimensional supra­molecular network.

## Related literature

For related magnesium(II) complexes with 1,10-phenanthroline and pyridine ligands, see: Halut-Desportes (1981 ▶); Hao *et al.* (2008 ▶); Zhang (2004 ▶); Zhang *et al.* (2010 ▶).
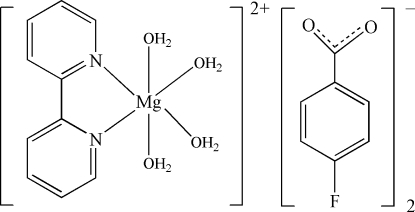


## Experimental

### 

#### Crystal data

[Mg(C_10_H_8_N_2_)(H_2_O)_4_](C_7_H_4_FO_2_)_2_
*M* *_r_* = 530.76
Orthorhombic, 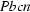

*a* = 27.911 (6) Å
*b* = 12.423 (3) Å
*c* = 7.5895 (15) Å
*V* = 2631.6 (10) Å^3^
*Z* = 4
Mo *K*α radiation
μ = 0.13 mm^−1^
*T* = 290 K
0.18 × 0.13 × 0.10 mm

#### Data collection

Rigaku R-AXIS RAPID diffractometer
Absorption correction: multi-scan (*ABSCOR*; Higashi, 1995) ▶ *T*_min_ = 0.979, *T*_max_ = 0.987
2316 measured reflections
2310 independent reflections
1741 reflections with *I* > 2σ(*I*)
*R*_int_ = 0.096

#### Refinement

*R*[*F* ^2^ > 2σ(*F* ^2^)] = 0.070
*wR*(*F* ^2^) = 0.207
*S* = 1.14
2310 reflections
156 parameters
H-atom parameters constrained
Δρ_max_ = 0.39 e Å^−3^
Δρ_min_ = −0.32 e Å^−3^

### 

Data collection: *RAPID-AUTO* (Rigaku, 1998) ▶; cell refinement: *RAPID-AUTO* ▶; data reduction: *CrystalStructure* (Rigaku/MSC, 2002) ▶; program(s) used to solve structure: *SHELXS97* (Sheldrick, 2008 ▶); program(s) used to refine structure: *SHELXL97* (Sheldrick, 2008 ▶); molecular graphics: *SHELXTL* (Sheldrick, 2008 ▶); software used to prepare material for publication: *SHELXL97*.

## Supplementary Material

Crystal structure: contains datablocks I, global. DOI: 10.1107/S1600536810047690/kp2287sup1.cif

Structure factors: contains datablocks I. DOI: 10.1107/S1600536810047690/kp2287Isup2.hkl

Additional supplementary materials:  crystallographic information; 3D view; checkCIF report

## Figures and Tables

**Table 1:** Hydrogen-bond geometry (Å, °)

*D*—H⋯*A*	*D*—H	H⋯*A*	*D*⋯*A*	*D*—H⋯*A*
O1—H1*A*⋯O4^i^	0.82	1.90	2.715 (4)	172
O1—H1*B*⋯O4^ii^	0.82	1.90	2.682 (4)	159
O2—H2*A*⋯O3^iii^	0.82	1.84	2.661 (3)	173
O2—H2*B*⋯O4^ii^	0.82	1.99	2.796 (5)	167
C3—H3⋯O3^iv^	0.93	2.55	3.257 (6)	133

Symmetry codes: (i) 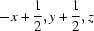; (ii) 
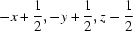
; (iii) 
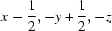
; (iv) 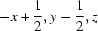.

**Fig. 1.**
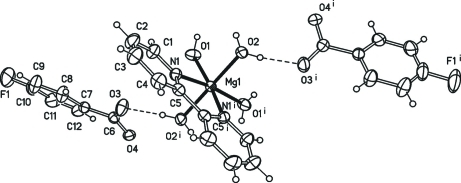
The molecule structure of the title compound showing the atom-labelling scheme. The octahedral [Mg(C10H8N2)(H2O)4]2+ complex cation is balanced by two fluorbenzoate anions. The two coordinated water molecules of the complex cation are hydrogen bonded to two anions. Displacement ellipsoids are drawn at the 40% probability level (symmetry code:(i)-x,y,-z+1/2).

**Fig. 2.**
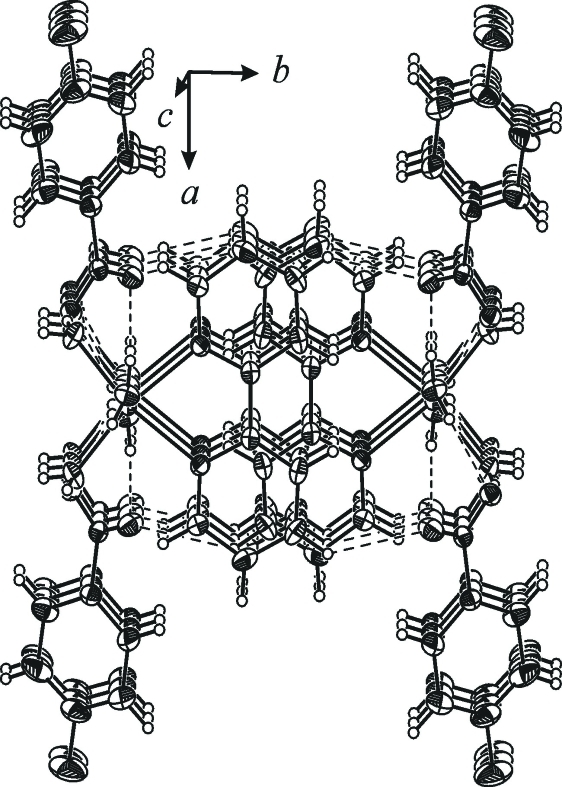
A packing diagram of the title complex, viewed down the c axis, The O—H···O and C—H···O hydrogen bonds(dashed lines) in the title compound.

## supplementary crystallographic information

### Comment

Magnesium(II) ions with 1.10-phenanthroline(phen) and pyridine(bipy) ligands can form tetraaqua(L)_n_Magnesium(II)(L = phen, n = 1; L = bipy, n = 2 ) complex cation ( Halut-Desportes, 1981, Hao,*et al.*, 2008, Zhang, 2004, Zhang, *et al.*,2010.) In this paper we report synthesis and structure of the title compound. The crystal structure of title compound consists of [Mg(H_2_O)_4_(2,2'-bpy)] ^2+^ complex cations and 4-fluorbenzoate anion (Fig. 1). the cation placed in special position on twofold axis which passes through Mg^II^ atom and middle C5—C5^i^ bond of 2,2'-bipy molecule; Symmetry code:(i)-x,y,-z+1/2. In the cation, the Mg^II^ atom is coordinated by two N atoms from one 2,2'-bipy ligands, four O atoms from four different water molecules, completing a distorted MgN_2_O_4_ octahedral geometry. The Mg—N bond length is 2.183 (3) Å and Mg—O bond lengths are 2.040 (2) and 2.061 (2)Å. The chelating bipy ligands exhibit nearly perfect planarity (r.m.s. deviations = 0.0061 Å). The mean interplanar distances of 3.8352 (3) Å between adjacent bipy ligands indicate π–π stacking interactions (very weak). The complex cations and 4-fluorbenzoate anins are connected via O—H···O and C—H···O hydrogen bonds (Table 1, Fig. 2) into a three-dimensional supramolecular network.

### Experimental

[Mg(OH)_2_.4MgCO_3_.4H_2_O} (0.4900 g, 1.00 mmol), 4-fluorbenzoate acid (0.0602 g, 0.43 mmol),2,2'-bipyridine (bipy) (0.0503 g, 0.32 mmol ), CH_3_OH/H_2_O (v/v = 1:2, 15 mL) were mixed and stirred for 2.0 h. Subsequently, the resulting cream suspension was heated in a 23 mL Teflon-lined stainless steel autoclave at 453 K for 5800 minutes. After the autoclave was cooled to room temperature according to the procedure for 2600 minutes, the solid was filtered off. The resulting filtrate was allowed to stand at room temperature, and slow evaporation for 2 months afforded colourless block single crystals.

### Refinement

C-bound H atoms were placed in calculated positions, with C—H = 0.93Å and *U*_iso_(H) = 1.2*U*_eq_(C), and were refined using the riding- model approximation. The H atoms of the water molecule were located in a difference Fourier map and refined with an O—H distance restraint of 0.82 (1) Å and *U*_iso_(H) = 1.5*U*_eq_(O).

### Crystal data

**Table:**

[Mg(C_10_H_8_N_2_)(H_2_O)_4_](C_7_H_4_FO_2_)_2_	*F*(000) = 1104
*M**_r_* = 530.76	*D*_x_ = 1.340 Mg m^−^^3^
Orthorhombic, *P**b**c**n*	Mo *K*α radiation, λ = 0.71073 Å
Hall symbol: -p 2n 2ab	Cell parameters from 665 reflections
*a* = 27.911 (6) Å	θ = 3.2–25.0°
*b* = 12.423 (3) Å	µ = 0.13 mm^−^^1^
*c* = 7.5895 (15) Å	*T* = 290 K
*V* = 2631.6 (10) Å^3^	Block, colourless
*Z* = 4	0.18 × 0.13 × 0.10 mm

### Data collection

**Table:**

Rigaku R-AXIS RAPID diffractometer	2310 independent reflections
Radiation source: fine-focus sealed tube	1741 reflections with *I* > 2σ(*I*)
graphite	*R*_int_ = 0.096
ω scans	θ_max_ = 25.0°, θ_min_ = 3.2°
Absorption correction: multi-scan (*ABSCOR*; Higashi, 1995)	*h* = −33→33
*T*_min_ = 0.979, *T*_max_ = 0.987	*k* = −14→14
2316 measured reflections	*l* = −8→9

### Refinement

**Table:**

Refinement on *F*^2^	Primary atom site location: structure-invariant direct methods
Least-squares matrix: full	Secondary atom site location: difference Fourier map
*R*[*F*^2^ > 2σ(*F*^2^)] = 0.070	Hydrogen site location: inferred from neighbouring sites
*wR*(*F*^2^) = 0.207	H-atom parameters constrained
*S* = 1.14	*w* = 1/[σ^2^(*F*_o_^2^) + (0.0879*P*)^2^ + 2.3043*P*] where *P* = (*F*_o_^2^ + 2*F*_c_^2^)/3
2310 reflections	(Δ/σ)_max_ < 0.001
156 parameters	Δρ_max_ = 0.39 e Å^−^^3^
0 restraints	Δρ_min_ = −0.32 e Å^−^^3^

### Special details

**Table:**

Geometry. All esds (except the esd in the dihedral angle between two l.s. planes) are estimated using the full covariance matrix. The cell esds are taken into account individually in the estimation of esds in distances, angles and torsion angles; correlations between esds in cell parameters are only used when they are defined by crystal symmetry. An approximate (isotropic) treatment of cell esds is used for estimating esds involving l.s. planes.
Refinement. Refinement of F^2^ against ALL reflections. The weighted R-factor wR and goodness of fit S are based on F^2^, conventional R-factors R are based on F, with F set to zero for negative F^2^. The threshold expression of F^2^ > 2sigma(F^2^) is used only for calculating R-factors(gt) etc. and is not relevant to the choice of reflections for refinement. R-factors based on F^2^ are statistically about twice as large as those based on F, and R- factors based on ALL data will be even larger.

### Fractional atomic coordinates and isotropic or equivalent isotropic displacement parameters (Å^2^)

**Table:**

	*x*	*y*	*z*	*U*_iso_*/*U*_eq_	
Mg1	0.0000	0.29487 (11)	0.2500	0.0347 (4)	
O1	0.05496 (9)	0.4005 (2)	0.2115 (3)	0.0536 (7)	
H1A	0.0576	0.4581	0.2632	0.080*	
H1B	0.0657	0.4124	0.1128	0.080*	
O2	−0.01046 (8)	0.2966 (2)	−0.0187 (3)	0.0509 (7)	
H2A	−0.0378	0.2905	−0.0568	0.076*	
H2B	0.0122	0.3254	−0.0688	0.076*	
O3	0.40257 (9)	0.2093 (2)	0.1617 (4)	0.0680 (9)	
O4	0.42874 (8)	0.0949 (2)	0.3633 (3)	0.0482 (7)	
N1	0.04711 (9)	0.1555 (2)	0.2250 (4)	0.0418 (7)	
F1	0.20665 (10)	0.0891 (3)	0.5077 (5)	0.1243 (14)	
C1	0.09402 (13)	0.1603 (3)	0.1902 (5)	0.0544 (10)	
H1	0.1090	0.2271	0.1896	0.065*	
C2	0.12112 (15)	0.0689 (4)	0.1551 (6)	0.0682 (12)	
H2	0.1537	0.0746	0.1312	0.082*	
C3	0.09930 (15)	−0.0285 (4)	0.1561 (6)	0.0689 (12)	
H3	0.1167	−0.0906	0.1313	0.083*	
C4	0.05129 (15)	−0.0346 (3)	0.1944 (6)	0.0586 (10)	
H4	0.0360	−0.1011	0.1969	0.070*	
C5	0.02579 (12)	0.0585 (3)	0.2293 (4)	0.0415 (8)	
C6	0.39599 (12)	0.1476 (3)	0.2874 (5)	0.0426 (8)	
C7	0.34578 (6)	0.1330 (2)	0.3515 (3)	0.0454 (8)	
C8	0.31097 (9)	0.20812 (19)	0.3063 (4)	0.0666 (12)	
H8	0.3192	0.2683	0.2400	0.080*	
C9	0.26384 (8)	0.1933 (2)	0.3604 (5)	0.0860 (16)	
H9	0.2405	0.2436	0.3302	0.103*	
C10	0.25153 (7)	0.1034 (3)	0.4595 (4)	0.0789 (14)	
C11	0.28634 (10)	0.0283 (2)	0.5047 (4)	0.0844 (17)	
H11	0.2781	−0.0319	0.5710	0.101*	
C12	0.33346 (9)	0.0431 (2)	0.4506 (4)	0.0663 (12)	
H12	0.3568	−0.0072	0.4808	0.080*	

### Atomic displacement parameters (Å^2^)

**Table:**

	*U*^11^	*U*^22^	*U*^33^	*U*^12^	*U*^13^	*U*^23^
Mg1	0.0374 (8)	0.0321 (8)	0.0346 (8)	0.000	−0.0008 (6)	0.000
O1	0.0681 (16)	0.0475 (14)	0.0450 (13)	−0.0212 (12)	0.0090 (12)	−0.0062 (12)
O2	0.0452 (14)	0.0697 (17)	0.0379 (13)	−0.0083 (12)	−0.0036 (10)	0.0040 (12)
O3	0.0505 (16)	0.076 (2)	0.0771 (19)	0.0115 (13)	0.0175 (14)	0.0332 (16)
O4	0.0473 (14)	0.0502 (15)	0.0472 (13)	0.0034 (11)	−0.0090 (11)	−0.0031 (11)
N1	0.0400 (16)	0.0406 (16)	0.0448 (15)	0.0030 (12)	−0.0008 (12)	−0.0011 (13)
F1	0.0611 (18)	0.169 (4)	0.143 (3)	−0.0280 (19)	0.0441 (18)	−0.016 (3)
C1	0.043 (2)	0.049 (2)	0.071 (3)	0.0030 (16)	0.0017 (18)	−0.0011 (19)
C2	0.047 (2)	0.072 (3)	0.085 (3)	0.015 (2)	0.010 (2)	−0.003 (2)
C3	0.066 (3)	0.052 (3)	0.089 (3)	0.024 (2)	0.006 (2)	−0.008 (2)
C4	0.070 (3)	0.036 (2)	0.070 (2)	0.0086 (18)	0.001 (2)	−0.0039 (18)
C5	0.0502 (19)	0.0371 (18)	0.0372 (17)	0.0012 (15)	−0.0021 (15)	−0.0001 (14)
C6	0.0430 (19)	0.0397 (19)	0.0451 (19)	0.0027 (15)	0.0018 (15)	−0.0051 (16)
C7	0.0430 (19)	0.050 (2)	0.0434 (18)	−0.0033 (16)	0.0019 (15)	−0.0017 (16)
C8	0.051 (2)	0.057 (2)	0.092 (3)	0.0051 (19)	0.013 (2)	0.007 (2)
C9	0.050 (3)	0.086 (4)	0.122 (4)	0.011 (2)	0.016 (3)	−0.004 (3)
C10	0.054 (3)	0.104 (4)	0.079 (3)	−0.017 (3)	0.023 (2)	−0.010 (3)
C11	0.073 (3)	0.112 (5)	0.068 (3)	−0.035 (3)	0.005 (2)	0.027 (3)
C12	0.059 (2)	0.081 (3)	0.059 (2)	−0.013 (2)	−0.0045 (19)	0.020 (2)

### Geometric parameters (Å, °)

**Table:**

Mg1—O1^i^	2.040 (2)	C2—H2	0.9300
Mg1—O1	2.040 (2)	C3—C4	1.373 (6)
Mg1—O2	2.061 (2)	C3—H3	0.9300
Mg1—O2^i^	2.061 (2)	C4—C5	1.384 (5)
Mg1—N1^i^	2.183 (3)	C4—H4	0.9300
Mg1—N1	2.183 (3)	C5—C5^i^	1.473 (7)
O1—H1A	0.8200	C6—C7	1.494 (4)
O1—H1B	0.8200	C7—C8	1.3900
O2—H2A	0.8201	C7—C12	1.3900
O2—H2B	0.8198	C8—C9	1.3900
O3—C6	1.238 (4)	C8—H8	0.9300
O4—C6	1.263 (4)	C9—C10	1.3900
N1—C1	1.337 (4)	C9—H9	0.9300
N1—C5	1.344 (4)	C10—C11	1.3900
F1—C10	1.317 (3)	C11—C12	1.3900
C1—C2	1.390 (6)	C11—H11	0.9300
C1—H1	0.9300	C12—H12	0.9300
C2—C3	1.355 (6)		
			
O1^i^—Mg1—O1	99.93 (16)	C2—C3—C4	119.3 (4)
O1^i^—Mg1—O2	91.62 (10)	C2—C3—H3	120.4
O1—Mg1—O2	87.60 (10)	C4—C3—H3	120.4
O1^i^—Mg1—O2^i^	87.60 (10)	C3—C4—C5	119.8 (4)
O1—Mg1—O2^i^	91.62 (10)	C3—C4—H4	120.1
O2—Mg1—O2^i^	178.78 (17)	C5—C4—H4	120.1
O1^i^—Mg1—N1^i^	92.57 (11)	N1—C5—C4	121.1 (3)
O1—Mg1—N1^i^	167.41 (12)	N1—C5—C5^i^	115.97 (18)
O2—Mg1—N1^i^	90.53 (10)	C4—C5—C5^i^	122.9 (2)
O2^i^—Mg1—N1^i^	90.44 (11)	O3—C6—O4	124.4 (3)
O1^i^—Mg1—N1	167.41 (12)	O3—C6—C7	117.7 (3)
O1—Mg1—N1	92.57 (11)	O4—C6—C7	117.9 (3)
O2—Mg1—N1	90.44 (11)	C8—C7—C12	120.0
O2^i^—Mg1—N1	90.53 (10)	C8—C7—C6	119.6 (2)
N1^i^—Mg1—N1	74.99 (15)	C12—C7—C6	120.4 (2)
Mg1—O1—H1A	124.2	C7—C8—C9	120.0
Mg1—O1—H1B	121.4	C7—C8—H8	120.0
H1A—O1—H1B	104.3	C9—C8—H8	120.0
Mg1—O2—H2A	118.7	C8—C9—C10	120.0
Mg1—O2—H2B	110.7	C8—C9—H9	120.0
H2A—O2—H2B	126.6	C10—C9—H9	120.0
C1—N1—C5	118.6 (3)	F1—C10—C11	120.4 (3)
C1—N1—Mg1	124.9 (2)	F1—C10—C9	119.6 (3)
C5—N1—Mg1	116.2 (2)	C11—C10—C9	120.0
N1—C1—C2	122.3 (4)	C10—C11—C12	120.0
N1—C1—H1	118.8	C10—C11—H11	120.0
C2—C1—H1	118.8	C12—C11—H11	120.0
C3—C2—C1	118.9 (4)	C11—C12—C7	120.0
C3—C2—H2	120.5	C11—C12—H12	120.0
C1—C2—H2	120.5	C7—C12—H12	120.0

Symmetry codes: (i) −*x*, *y*, −*z*+1/2.

### Hydrogen-bond geometry (Å, °)

**Table:**

*D*—H···*A*	*D*—H	H···*A*	*D*···*A*	*D*—H···*A*
O1—H1A···O4^ii^	0.82	1.90	2.715 (4)	172
O1—H1B···O4^iii^	0.82	1.90	2.682 (4)	159
O2—H2A···O3^iv^	0.82	1.84	2.661 (3)	173
O2—H2B···O4^iii^	0.82	1.99	2.796 (5)	167
C3—H3···O3^v^	0.93	2.55	3.257 (6)	133

Symmetry codes: (ii) −*x*+1/2, *y*+1/2, *z*; (iii) −*x*+1/2, −*y*+1/2, *z*−1/2; (iv) *x*−1/2, −*y*+1/2, −*z*; (v) −*x*+1/2, *y*−1/2, *z*.
